# Supplementary material for: A singular PpaA/AerR-like protein in Rhodospirillum rubrum rules beyond the boundaries of photosynthesis in response to the intracellular redox state
Source: mSystems. 2023 Dec 6;8(6):e00702-23. doi: 10.1128/msystems.00702-23 (PMC10734443; doi:10.1128/msystems.00702-23)
Supplement: Supplemental figures and tables — Fig. S1 to S3; Tables S1 to S3. [file msystems.00702-23-s0001.pdf]

SUPPLEMENTARY MATERIAL FOR RESEARCH ARTICLE

A singular PpaA/AerR-like protein *in Rhodospirillum rubrum* rules beyond the boundaries of photosynthesis in response to the intracellular redox state

Manuel S. Godoy [1, 2], Santiago R. de Miguel [1, 2], M. Auxiliadora Prieto[1, 2]**

[1]Polymer Biotechnology Lab, Biological Research Centre Margarita Salas, Spanish National Research Council (CIB-CSIC), Madrid, Spain.

[2]Interdisciplinary Platform for Sustainable Plastics towards a Circular Economy-CSIC (SusPlast-CSIC), Madrid, Spain.

*corresponding authors: auxi@cib.csic.es, msgodoy@cib.csic.es

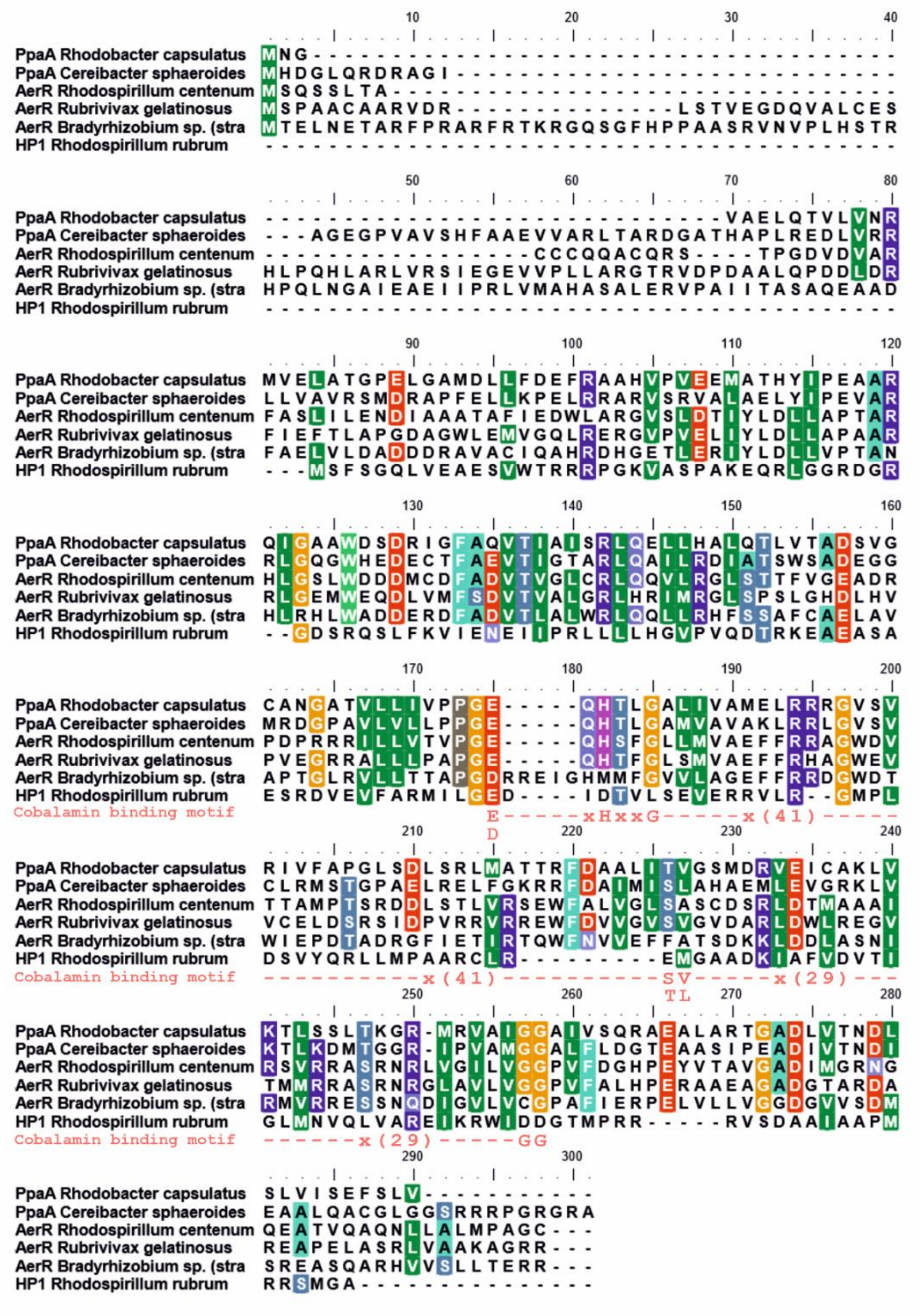

Fig. S1: Multiple sequence alignment of PpsR antirepressors. The sequences of the representative PpsR-related proteins AerR and PpaA, as well as the HP1 sequence, were aligned using the ClustalW algorithm. The amino acids conserved within 70% of the sequences were colored. The cobalamin binding motif (in red) admits more than one amino acid in some positions (1, 2) The Uniprot accession numbers of the proteins used for the analysis are: PpaA *Rhodobacter capsulatus* (D5ANS8); PpaA *Cereibacter sphaeroides* (Q3J178); AerR *R. centenum* (B6ITX0); AerR *Rubrivivax gelatinosus* (I0HUJ6); AerR *Bradyrhizobium* sp. (Q6A566); HP1 *R. rubrum* (Q2RWR6).

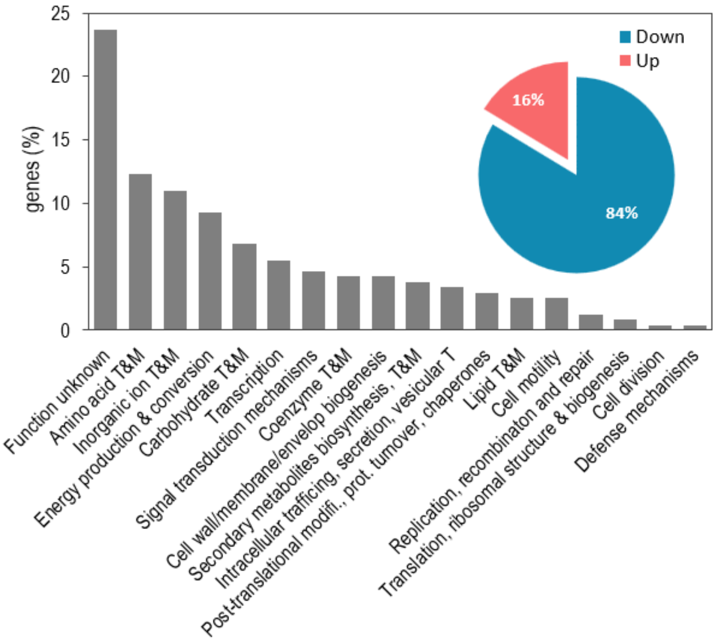

Fig. S2: COG analysis of significantly down- and up-regulated genes. The database of Clusters of Orthologous Groups of proteins (COGs) was used to rationally classify genes whose expression is affected in ΔA0625 strain compared to the wild type, under microaerobic conditions. The average in the column graph represents the proportion of genes (from the whole genome of *R. rubrum*) grouped in each category whose expression was significantly affected. In the pie chart the proportion up- and down- regulated genes can be seen. T, transport; M, metabolism

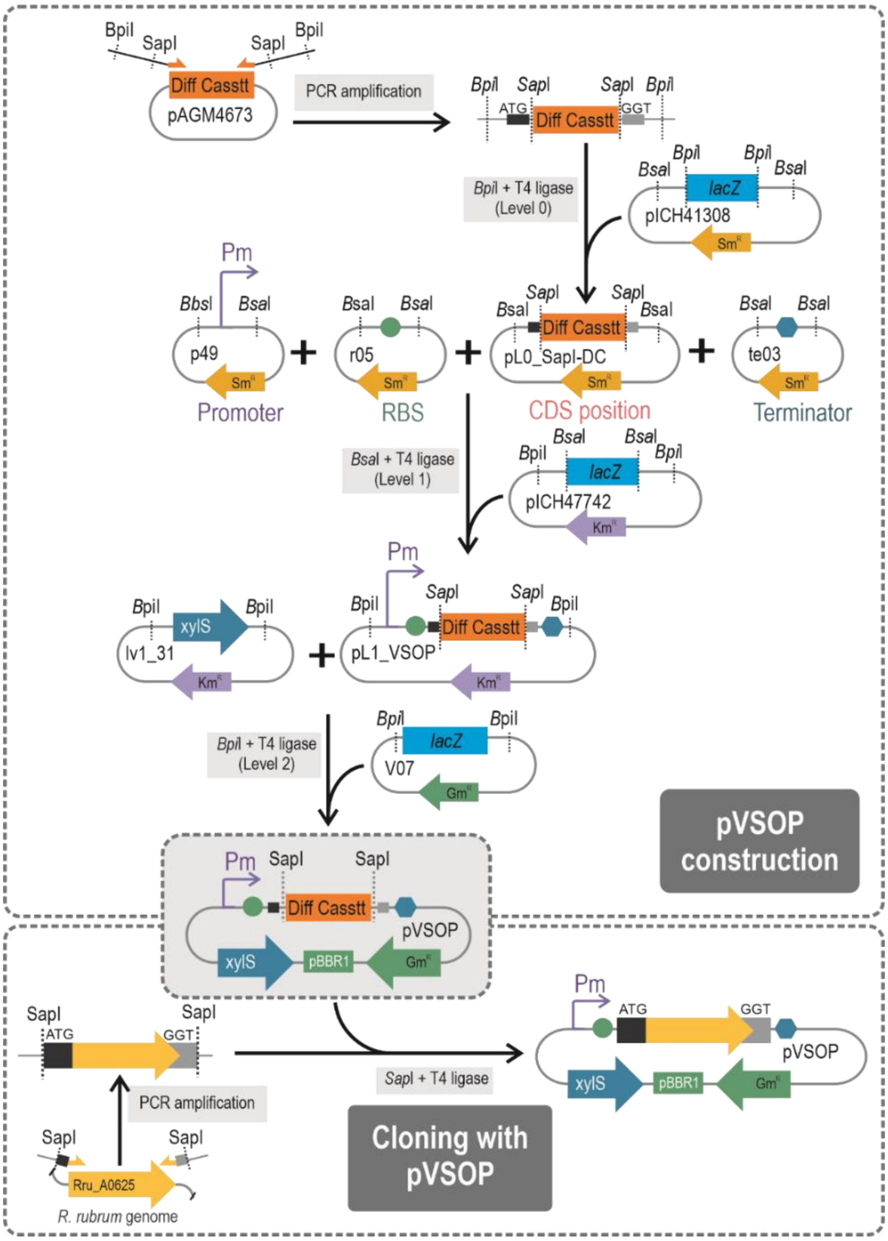

Fig. S3. Scheme of the procedure for creating the expression plasmid pVSOP and its subsequent utilization in cloning coding sequences. For the construction of plasmid pVSOP, plasmids from Golden Standard assembly kit (3) and Marillonet collection (4) were also used, following the conventional procedure of Golden Gate (GG) reactions and increasing complexity on each cloning level (0, 1 and 2). The expression plasmid pVSOP allowed the cloning of Open Reading Frames (ORFs) also by means of GG using restriction enzyme *Sap*I. All the plasmids have a pBBR replicon.

Table S1. Plasmids used in this work. BkB, backbone; Compl, complementation; DC, differentiation cassette; RBS, ribosom binding site; TU, transcription unit.

Name	Relevant feature	Use	Reference
pSEVA231	EcoRi/HindIII restriction sites. Km^R. Ori pBBR1	$HP1^{wt/M}$ cloning	(3)
pSEVA_HP1	Contained 1.8 kb including Rru_A0625, and Rru_A0625b from the wild type strain	Compl. of strain ΔA0625	This work
pAGM4673	*crtEYIB* genes from *Pantoea ananatis*	PCR template for SapI-DC	(4)
pICH41308	BpiI cloning/BsaI subcloning. Stp^R,	pVSOP assembly (Level 0, CDS cloning BkB)	(4)
pL0_SapI-DC	DC containing *crtEYIB* from *P. ananatis*, and SapI cloning site. Sm^R	pL1_VSOP assembly (Level 0 – CDS position)	This work
p49	Pm inducible promoter (Pm-XylS system inducible by 3MB). Amp^R	pL1_VSOP assembly (Level 0 – Prom. position)	(5)
r05	Strong bicistronic RBS. Amp^R	pL1_VSOP assembly (Level 0 – RBS position)	(5)
te03	rpoC transcription terminator. Sm^R	pL1_VSOP assembly (Level 0 – Term position)	(5)
pICH47742	BsaI cloning/BpiI subcloning. Am^R,	pVSOP assembly (Level 1, 2nd TU cloning BkB)	(4)
pL1_VSOP	Pm, BC12, SapI-DC, *rpoC* Term. Gm^R.	pVSOP assembly (Level 1 – 2nd TU)	This work
lv1_31	XylS transcription factor. Km^R	pVSOP assembly (Level 1 – 1st TU)	(5)
V07	BpiI cloning. Gm^R.	pVSOP assembly (Level 2, 2-TU cloning BkB)	(5)
pVSOP	Contains XylS, SapI cloning site. Produces orange colonies. Ori pBBR1	CDS cloning vector for 3MB-induced expression	This work
$pVSOP\_A0625^{S1}$	Expression of Rru_A0625	Compl. of strain ΔA0625	This work
$pVSOP\_A0625^{B}$	Expression of Rru_A0625b	Compl. of strain ΔA0625	This work
pVSOP_Empty	Control plasmid expressing MSGAG amino acids	Compl. of strain ΔA0625	This work
pK18*mobsacB*	Plasmid containing sacB gene for negative selection after double homologous recombination	Construction of plasmid pK18msg	Schäfer 1994
pK18msg	Derivative of pK18*mobsacB* with modified cloning site for 'Golden Gate' assembly (*Bpi*I, *Bsa*I, *Aer*I)	Construction of strain ΔA0625	This work
pK18_ΔA0625	Homologous regions flanking Rru_A0625	Construction of strain ΔA0625	This work

Table S2. Oligonucleotides used in this work. HR, homologous region flanking the gene to be deleted (A, upstream; B, downstream); DC, differentiation cassette.

Name	Sequence	Use	Restriction site
O_43	TTT**GAATTC**GCAGGTGGTCTTCGAGACCCCTGGAGATCCTGGGTCTC TGGAAGACCGCACCTGCTC**AAGCTT**ATC	MCS of pK18msg	EcoRI, HindIII
O_44	GAT**AAGCTT**GAGCAGGTGCGGTCTTCCAGAGACCCAGGATCTCCAGG GGTCTCGAAGACCACCTGC**GAATTC**AAA	MCS of pK18msg	EcoRI, HindIII
O_77	TT**GGATCC**TCCTGTCGATGTTGAGCG	$pSEVA\_HP1^{wt/M}$, check ΔA0625	BamHI
O_78	TT**GTCGAC**ATCGAGACCTTCAAGGACG	$pSEVA\_HP1^{wt/M}$, check ΔA0625	BamHI
O_81	TTA**GAAGAC**AACAGGCACCACCACCGAGGCGTT	HR A of Rru_A0625	BbsI
O_82	ATC**GAAGAC**CGCCTCCGTCCC	HR A of Rru_A0625	BbsI
O_83	TTT**GAAGAC**ACGAGGGCGTCGGTCTATGGGGGC	HR B of Rru_A0625	BbsI
O_84	TTT**GAAGAC**AAGGTGTTCGTCTGGAAATCGCCC	HR B of Rru_A0625	BbsI
O_207	TTT**GCTCTTC**GATGATCCGGGACGGAGGCGGTC	Clone Rru_A0625 in pVSOP	SapI
O_211	TTT**GCTCTTC**TACCGGCCCCCATAGACCGACGC	Clone Rru_A0625 in pVSOP	SapI
O_225	TTT**GCTCTTC**GATGGCGTCCGGGAGGGA	Clone Rru_A0625b in pVSOP	SapI
O_226	TTT**GCTCTTC**TACCGACCGCTTTTGTCGGAAT	Clone Rru_A0625b in pVSOP	SapI
O_167	TAT**GAAGAC**GTAATGT**GAAGAGC**CAGTGGTATGGGGTACCGCA	Clone DC	BbsI, SapI
O_168	TAT**GAAGAC**TTAAGCTTTAT**GAAGAGC**CACTTGAGTGGTTTTTAAT	Clone DC	BbsI, SapI

Table S3. Transcriptomic analysis of ΔA0625 vs wild type strains. Genes significantly affected by Rru_A0625 deletion are shown. Loci names written in blue have a BH p-value>0.05 but were still included in this analysis since they are placed within a Transcription Cluster (TC) together with genes significantly affected (BH p-value<0.05). The TC are highlighted in light green or violet. The nomenclature of loci used was the most frequent in literature, corresponding to assembly ASM1308v1 (accession number). Loci RS03635, RS06115, RS09900, RS11125 were only annotated in the assembly used to map the reads in the transcriptome (ASM1913455v1). **Abbreviations:** Aac Met, Amino acid metabolism; Cat Act, Catalitic activity; CH Met & Energy, Carbohydrate metabolism and energy production/consumption; dep, dependent; dh, dehydrogenase; Fe:proChlide, Ferredoxin:protochlorophyllide; HP, Hypothetical protein; Lip Met, Lipid Metabolism; Mg-PMEOC, Mg-protoporphyrin IX monomethyl ester anaerobic oxidative cyclase; Nit Met, Nitrogen Metabolism; ox, oxidase; P, phospahate; Pat & Def, Pathogenity and defence; prot, protein; PS, photosynthetic; PSA, Photosynthetic Aparatus; RC, Reaction Center; red, reductase; Resp, Response; Sig Transd, Signal transduction; Stress Rsp, Stress response; su., subunit; Sug &PScc, Sugar and Polysaccharide metabolism; T&M, Transport and metabolism; unk, unknown.

Fold change

-160 -120 -80 -20 -10 0 5 10

Locus	Fold Change*	Description	Gene	Process
Rru_B0037	-3	Hemolysin-type calcium-binding region	-	Pat & Def
Rru_A0073	-2	HPF/raia family ribosome-associated prot	-	Regulation
Rru_A0106	2	Tetratricopeptide repeat prot	-	unk
Rru_A0109	-2	Type II and III secretion system prot	-	Motility
Rru_A0112	-2	Prepilin-type N-terminal cleavage/methylation dom-containing prot	-	Motility
Rru_A0119	-9	DUF1328 domain-containing prot	-	unk
Rru_A0121	-2	HP	-	unk
Rru_A0122	-3	I78 family peptidase inhibitor	-	Pat & Def
Rru_A0156	-2	Hlyd family secretion prot	-	Pat & Def
Rru_A0160	-7	Usg prot	-	Aac Met
Rru_A0184	-2	RAMP superfamily CRISPR-associated prot	-	Pat & Def
Rru_A0185	-2	HP	-	Pat & Def
Rru_A0200	-2	Restriction endonuclease	-	Pat & Def
Rru_A0202	-4	VTT domain-containing prot	-	Lip Met
Rru_A0223	-3	Polyphosphate--AMP phosphotransferase	-	CH Met & Energy
Rru_A0261	2	DUF2971 domain-containing prot	-	unk
Rru_A0269	-4	Ompa family prot	-	unk
Rru_A0270	-5	Co_2+/Mg_2+ efflux prot	*apaG*	Ion Balance
Rru_A0338	-2	Nuclear transport factor 2 family prot	-	unk
Rru_A0385	-2	Gamma-glutamyltransferase	*ggt*	Aac Met
Rru_A0420	-3	PRC-barrel domain-containing prot	-	PSA
Rru_A0421	-6	HP	-	unk
Rru_A0422	-7	HP	-	unk
Rru_A0423	-4	HP	-	unk
Rru_A0424	-4	DUF3096 domain-containing prot		unk
Rru_A0430	-5	Yihy/virulence factor brkb family prot	-	Pat & Def
Rru_A0442	2	FAD-binding oxidored	-	CH Met & Energy
Rru_A0462	-5	NAD-dep succinate-semialdehyde dh	-	CH Met & Energy
Rru_A0463	-3	Alanine racemase	-	Aac Met
Rru_A0485	-2	Magnesium chelatase atpase su. I	*bchI*	PSA
Rru_A0488	-2	Tonb-dep receptor	-	PSA
Rru_A0492	-3	Adenosylcobinamide amidohydrolase	-	PSA
Rru_A0493	-4	Phytoene desaturase	-	PSA
Rru_A0494	-5	Phytoene/squalene synthase family prot	-	PSA
Rru_A0495	-7	HP	-	unk
Rru_A0496	-3	Crp/Fnr family transcriptional regulator	-	Regulation
Rru_A0501	-2	Transposase	-	Pat & Def
Rru_A0502	-2	AAA family atpase	-	unk
Rru_A0505	-5	Glycogen debranching prot	*glgX*	S&PSc
Rru_A0511	-2	DEAD/DEAH box helicase	-	Regulation
Rru_A0512	-2	DUF1837 domain-containing prot	-	unk
Rru_A0586	2	Co-chaperone groes	*groES*	Stress Rsp
Rru_A0587	2	Chaperonin groel	*groL*	Stress Rsp

Locus	Fold Change*	Description	Gene	Process
Rru_A0598	3	Psts family phosphate ABC transporter substrate-binding prot	-	Ion Balance
Rru_A0599	3	Phosphate ABC transporter permease su. Pstc	*pstC*	Ion Balance
Rru_A0613	-2	Bacteriochlorophyll 4-vinyl red	*bchJ*	PSA
Rru_A0614	-19	Putative PS complex assembly prot	*puhE*	PSA
Rru_A0615	-15	Putative PS complex assembly prot	*puhC*	PSA
Rru_A0616	-15	PH domain-containing prot	-	unk
Rru_A0617	-29	PS RC su. H	*puhA*	PSA
Rru_A0618	-24	BCD family MFS transporter	-	PSA
Rru_A0619	-30	Mg-protoporphyrin IX methyltransferase	*bchM*	PSA
Rru_A0620	-26	Fe:prochlide red (ATP-dep) Fe-S ATP-binding prot	*bchL*	PSA
Rru_A0621	-44	Mg-chelatase su. H	-	PSA
Rru_A0622	-28	Fe:prochlide red (ATP-dep) su. B	*bchB*	PSA
Rru_A0623	-21	Fe:prochlide red (ATP-dep) su. N	-	PSA
Rru_A0624	-23	2-vinyl bacteriochlorophyllide hydratase	*bchF*	PSA
Rru_A0627	-2	Chlorophyll synthase chlg	*chlG*	PSA
Rru_A0628	-3	BCD family MFS transporter	-	PSA
Rru_A0629	-6	Geranylgeranyl di-P red	-	PSA
Rru_A0630	-4	HP	-	unk
Rru_A0631	-3	Sensor domain-containing diguanylate cyclase	-	Regulation
Rru_A0665	-2	Resp regulator	-	Regulation
Rru_A0666	-5	CHASE3 domain-containing prot	-	Regulation
RS03635	4	HP	-	unk
Rru_A0702	2	Molybdenum ABC transporter ATP-binding prot	*modC*	Ion Balance
Rru_A0703	2	Molybdate ABC transporter permease su.	*modB*	Ion Balance
Rru_A0704	2	Molybdate ABC transporter substrate-binding prot	*modA*	Ion Balance
Rru_A0721	-4	Chrr family anti-sigma-E factor	-	Regulation
Rru_A0726	-6	MFS transporter	-	S&PSc
Rru_A0727	-5	DUF3833 domain-containing prot	-	unk
Rru_A0729	-4	SDR family NAD(P)-dep oxidored	-	unk
Rru_A0730	-7	DUF6134 family prot	-	unk
Rru_A0745	-2	Phosphoenolpyruvate--prot phosphotransferase	*ptsP*	S&PSc
Rru_A0759	-3	Aldo/keto red	-	CH Met & Energy
Rru_A0767	3	ABC transporter ATP-binding prot	-	Aac Met
Rru_A0792	4	Serine O-acetyltransferase	*cysE*	Aac Met
Rru_A0797	-2	MFS transporter	-	Aac Met
Rru_A0798	-5	Aminotransferase class I/II-fold pyridoxal P-dep	-	PSA
Rru_A0799	-2	ABC transporter substrate-binding prot	-	Aac Met
Rru_A0804	-4	Lysr family transcriptional regulator	-	Regulation
Rru_A0811	-5	Ribbon-helix-helix domain-containing prot	-	Regulation
Rru_A0838	-3	Tonb-dep receptor	-	Ion Balance
Rru_A0884	3	Fecr domain-containing prot	-	Ion Balance
Rru_A0885	-3	Tonb-dep siderophore receptor	-	Ion Balance
Rru_A0898	-3	Hpch/hpai aldolase/citrate lyase family prot	-	CH Met & Energy
Rru_A0899	-4	Cysteine protease stip family prot	-	Regulation
Rru_A0924	-4	Class I mannose-6-P isomerase	-	S&PSc
Rru_A0925	-3	Glycosyltransferase	-	S&PSc
Rru_A0973	-4	Ferritin-like domain-containing prot	-	Ion Balance
Rru_AR0025	-3	Trna-Glu	-	Translation
Rru_A0978	3	Marr family winged helix-turn-helix transcriptional regulator.	-	Regulation
Rru_A0979	4	ABC transporter substrate-binding prot	-	Aac Met
Rru_A0980	4	ABC transporter permease	-	Aac Met
Rru_A0981	4	ABC transporter permease	-	Aac Met
Rru_A0987	-3	Outer membrane prot	-	unk
Rru_A1010	-9	Nitrogenase iron prot	*nifH*	Nit Met
Rru_A1011	-7	Nitrogenase molybdenum-iron prot alpha chain	*nifD*	Nit Met
Rru_A1012	-9	Nitrogenase molybdenum-iron prot su. Beta	*nifK*	Nit Met
Rru_A1030	-2	Ethanolamine permease	*eat*	Aac Met
Rru_A1061	3	Ribbon-helix-helix domain-containing prot	-	Regulation
Rru_A1062	2	Type II toxin-antitoxin system vapc family toxin	-	Regulation
Rru_A1119	-3	Nnrs family prot	-	Ion Balance
Rru_A1122	-3	MBL fold metallo-hydrolase	-	unk

Locus	Fold Change*	Description	Gene	Process
Rru_A1129	3	Ammonium transporter	*amtB*	Nit Met
Rru_A1130	3	P-II family nitrogen regulator	*glnJ*	Nit Met
Rru_A1169	-2	Hydrogenase nickel incorporation prot hypb	*hypB*	CH Met & Energy
RS06115	-3	HP	-	unk
Rru_A1171	-2	Rrf2 family transcriptional regulator	-	PSA
Rru_A1175	-5	Csbd family prot	-	Stress Rsp
Rru_A1273	2	Alpha/beta hydrolase	-	Lip Met
Rru_A1282	3	Resp regulator	-	Regulation
Rru_A1285	-2	Trypsin-like peptidase domain-containing prot	-	CH Met & Energy
Rru_A1303	2	Imidazolonepropionase	*hutI*	Aac Met
Rru_A1304	2	Urocanate hydratase	*hutU*	Aac Met
Rru_A1350	-3	Cation:proton antiporter	-	Ion Balance
Rru_A1352	-8	DUF3008 family prot	-	unk
Rru_A1353	-5	DUF4142 domain-containing prot	-	unk
Rru_A1354	-5	DUF2934 domain-containing prot	-	unk
Rru_A1355	-4	Ferritin-like domain-containing prot	-	Ion Balance
Rru_A1356	-8	Catalase	-	Stress Rsp
Rru_A1367	3	SIS domain-containing prot	-	S&PSc
Rru_A1453	-2	Tail fiber prot	-	Pat & Def
Rru_A1454	-2	Tail fiber prot	-	Pat & Def
Rru_A1455	-3	Tail fiber prot	-	Pat & Def
Rru_A1460	-2	Asparagine synthase-related prot	-	Aac Met
Rru_A1461	-2	2-isopropylmalate synthase	*leuA*	Aac Met
Rru_A1497	-2	Cyclic peptide export ABC transporter	-	unk
RS07775	-14	DUF3309 family prot	-	unk
Rru_A1501	-7	BON domain-containing prot	-	Stress Rsp
Rru_A1502	-4	HP	-	unk
Rru_A1510	-7	HP	-	unk
Rru_A1510	-7	PRC-barrel domain-containing prot	-	PSA
Rru_A1537	-4	HP	-	unk
Rru_A1624	-4	HP	-	unk
Rru_A1661	-3	Extensin family prot	-	unk
Rru_AR0033	2	Trna-Ser	-	Translation
Rru_A1746	-2	Branched-chain amino acid ABC transporter substrate-binding prot	-	Aac Met
Rru_A1755	-5	HP	-	unk
Rru_A1782	-5	Superoxide dismutase family prot	-	Stress Rsp
Rru_A1785	-4	HP	-	unk
Rru_A1840	-3	Penicillin acylase family prot	-	Stress Rsp
Rru_A1847	-6	Transglutaminase family prot	-	Regulation
Rru_A1863	-5	Glutathione perox	-	Stress Rsp
RS09900	-6	HP	-	unk
Rru_A1936	-2	L,D-transpeptidase family prot	-	S&PSc
Rru_A1943	-2	Propionyl-coa carboxylase	-	Lip Met
Rru_A1974	-3	Type II toxin-antitoxin system hipa family toxin	-	Regulation
Rru_A1975	-4	Helix-turn-helix domain-containing prot	-	Regulation
Rru_A1976	-7	Phenylacetate-coa ligase	-	Aac Met
Rru_A1989	-3	N-acetylglutaminylglutamine amidotransferase	-	Aac Met
Rru_A1990	-5	HP	-	Motility
Rru_A1991	-6	TIGR02587 family membrane prot	-	Motility
Rru_A2005	-2	Acyl-coa dh	-	Lip Met
Rru_A2006	-3	Coa transferase	-	CH Met & Energy
Rru_A2017	-4	HP	-	unk
Rru_A2020	-3	Type II toxin-antitoxin system rele/pare family toxin.	-	Regulation
Rru_A2037	-3	Asma-like C-terminal domain-containing prot	-	Lip Met
Rru_A2049	-2	Tetr/acrr family transcriptional regulator	-	Regulation
Rru_A2050	-2	Efflux RND transporter periplasmic adaptor su.	-	Pat & Def
Rru_A2051	-2	Efflux RND transporter permease su.	-	unk
Rru_A2062	-6	HP	-	unk
Rru_A2091	-11	HP	-	unk
Rru_A2092	-8	HP	-	unk
Rru_A2099	-5	Autotransporter assembly complex prot	-	Pat & Def

Locus	Fold Change*	Description	Gene	Process
Rru_A2125	-2	Glycosyltransferase family 39 prot	-	S&PSc
RS11125	-2	HP	-	unk
Rru_A2176	3	ABC transporter permease	-	Aac Met
Rru_A2177	3	ABC transporter permease	-	Aac Met
Rru_A2178	3	ABC transporter substrate-binding prot	-	Aac Met
Rru_A2208	-4	Ribbon-helix-helix domain-containing prot	-	Regulation
Rru_A2261	-5	HP	-	unk
Rru_A2281	-10	Nitrogen fixation prot	*nifQ*	Nit Met
Rru_A2282	-10	Ferredoxin III, nif-specific	*fdxB*	Nit Met
Rru_A2283	-11	Nifx-associated nitrogen fixation prot	-	Nit Met
Rru_A2284	-7	Nitrogen fixation prot	*nifX*	Nit Met
Rru_A2285	-4	Nitrogenase iron-molybdenum cofactor biosynthesis	*nifN*	Nit Met
Rru_A2294	-8	Alpha-amylase family glycosyl hydrolase	-	S&PSc
Rru_A2295	-4	Malto-oligosyltrehalose trehalohydrolase	*treZ*	S&PSc
Rru_A2319	-2	2-methylcitrate synthase	*prpC*	CH Met & Energy
Rru_A2366	2	Cache domain-containing prot	-	unk
Rru_A2384	-2	ABC transporter permease	-	Aac Met
Rru_A2385	-4	ABC transporter substrate-binding prot	-	unk
Rru_A2404	-3	Phosphoribulokinase	-	S&PSc
Rru_A2405	-3	Transketolase	*tkt*	S&PSc
Rru_A2407	2	GNAT family N-acetyltransferase	-	Regulation
Rru_A2454	2	HP	-	unk
Rru_A2485	-4	Trehalose-6-P synthase	-	S&PSc
Rru_A2508	-8	5-aminolevulinate synthase	*hemA*	PSA
Rru_A2519	-5	Yqae/Pmp3 family membrane prot	-	unk
Rru_A2549	-5	Trehalose-phosphatase	*otsB*	S&PSc
Rru_A2576	-3	1,4-alpha-glucan branching prot glgb	*glgB*	S&PSc
Rru_A2577	-2	Glycogen debranching prot glgx	*glgX*	S&PSc
Rru_A2586	-4	HP	-	unk
Rru_A2589	-3	DUF4055 domain-containing prot	-	unk
Rru_A2730	-5	HP	-	unk
Rru_A2731	-4	Exopolysaccharide biosynthesis prot	-	S&PSc
Rru_A2741	-2	Polysaccharide biosynthesis prot	-	S&PSc
Rru_A2742	-2	Peptide transporter	-	unk
Rru_A2743	-3	Radical SAM prot	-	CH Met & Energy
Rru_A2744	-3	Wbqc family prot	-	CH Met & Energy
Rru_A2804	3	ABC transporter ATP-binding prot	-	unk
Rru_A2805	3	ABC transporter permease	-	unk
Rru_A2806	3	ABC transporter permease	-	unk
Rru_A2807	4	Fe-S-containing prot	-	unk
Rru_A2817	3	Phasin family prot	-	S&PSc
Rru_A2821	-2	Flagellar biosynthesis prot flhb	*flhB*	Motility
Rru_A2870	-3	Terc family prot	-	Stress Rsp
Rru_A2910	-3	Mlad family prot	-	Lip Met
Rru_A2974	-133	PS RC su. M	*pufM*	PSA
Rru_A2975	-157	PS RC su. L	*pufL*	PSA
Rru_A2976	-93	Light-harvesting prot	-	PSA
Rru_A2977	-108	Light-harvesting prot	-	PSA
Rru_A2978	-128	Chlorophyllide a red su. Z	*bchZ*	PSA
Rru_A2979	-58	Chlorophyllide a red su. Y	*bchY*	PSA
Rru_A2980	-57	Chlorophyllide a red iron prot su. X	-	PSA
Rru_A2981	-52	Chlorophyll synthesis pathway prot	*bchC*	PSA
Rru_A2982	-30	O-methyltransferase	*crtF*	PSA
Rru_A2983	-18	Polyprenyl synthetase family prot	-	PSA
Rru_A2984	-19	Phytoene desaturase family prot	*crtD*	PSA
Rru_A2985	-22	Carotenoid 1,2-hydratase	*crtC*	PSA
Rru_A2986	-4	FAD-dep tricarballylate dh	*tcuA*	Pat & Def
Rru_A2994	-3	AAA family atpase	-	unk
Rru_A2995	-6	HP	-	unk
Rru_A3016	-2	Phosphotransferase	-	CH Met & Energy
Rru_A3018	-8	General stress prot	-	Stress Rsp

Locus	Fold Change*	Description	Gene	Process
Rru_A3019	-10	General stress prot	-	Stress Rsp
Rru_A3020	-8	Spy/cpxp family prot refolding chaperone	-	Stress Rsp
Rru_A3038	-5	Tellurite resistance terb family prot	-	Stress Rsp
Rru_A3039	-3	Resp regulator	-	Regulation
Rru_A3044	-7	Yqci/ycgg family prot	-	unk
Rru_A3045	-6	Urea carboxylase-associated family prot	-	Nit Met
Rru_A3121	-2	Amidotransferase 1, exosortase A system-associated	-	Aac Met
Rru_A3122	-3	Acyl carrier prot	-	Lip Met
Rru_A3123	-2	Hydrolase 2, exosortase A system-associated	-	S&PSc
Rru_A3143	-4	Aldolase	-	S&PSc
Rru_A3171	-6	HP	-	unk
Rru_A3181	-6	HP	-	unk
Rru_A3189	-5	HP	-	unk
Rru_A3206	-3	Resp regulator	-	Regulation
Rru_A3207	-3	DUF423 domain-containing prot	-	unk
Rru_A3219	2	Iron ABC transporter substrate-binding prot	-	Ion Balance
Rru_A3272	-2	DMT family transporter	-	Stress Rsp
Rru_A3285	-3	Resp regulator	-	Regulation
Rru_A3286	-4	HP	*nepR*	Regulation
Rru_A3287	-4	Sigma-70 family RNA polymerase sigma factor	-	Regulation
Rru_A3297	2	Azlc family ABC transporter permease	-	Aac Met
Rru_A3304	-5	ABC transporter ATP-binding prot	-	Aac Met
Rru_A3305	-3	ABC transporter ATP-binding prot	-	Aac Met
Rru_A3306	-3	Branched-chain amino acid ABC transporter permease	-	Aac Met
Rru_A3307	-2	Branched-chain amino acid ABC transporter permease	-	Aac Met
Rru_A3349	-2	Gyri-like domain-containing prot	-	Regulation
Rru_A3417	2	ABC transporter substrate-binding prot	-	Aac Met
Rru_A3466	5	Ornithine--oxo-acid transaminase	*rocD*	Aac Met
Rru_A3467	7	Arginine deiminase-related prot	-	Aac Met
Rru_A3469	-5	HP	-	unk
Rru_A3520	-6	AI-2E family transporter	-	Pat & Def
Rru_A3528	-5	HP	-	unk
Rru_A3534	-5	HP	-	unk
Rru_A3548	-13	Mg-PMEOC	*bchE*	PSA
Rru_A3549	-6	Cobyric acid synthase	-	PSA
Rru_A3550	-9	Cobyric acid synthase	-	PSA
Rru_A3551	-7	Aldo/keto red	-	PSA
Rru_A3554	2	Molecular chaperone dnaj	*dnaJ*	Stress Rsp
Rru_A3618	-2	Protoporphyrinogen ox hemj	*hemJ*	PSA
Rru_A3645	-3	NAD-dep prot deacylase	*cobB*	Regulation
Rru_A3662	-3	HP	-	unk
Rru_A3667	-5	SDR family oxidored.	-	Lip Met
Rru_A3671	-2	Prot translocase su. Secd	*secD*	Pat & Def
Rru_A3675	-3	Glutathione S-transferase	-	Stress Rsp
Rru_A3676	-2	TRAP transporter large permease su.	-	CH Met & Energy
Rru_A3702	2	Metal ABC transporter ATP-binding prot	-	Ion Balance
Rru_A3763	2	Trna (adenosine(37)-N6)-threonylcarbamoyltransferase complex dimerization su. Type 1 tsab	*tsaB*	Translation
Rru_A3766	-2	Mucr family transcriptional regulator	-	Regulation

REFERENCES

1. Matthews RG. 2009. Cobalamin- and corrinoid-dependent enzymes. Met Ions Life Sci 6:53–114.
2. Cheng Z, Li K, Hammad LA, Karty JA, Bauer CE. 2014. Vitamin B12 regulates photosystem gene expression via the CrtJ antirepressor AerR in Rhodobacter capsulatus. Mol Microbiol 91:649–664.
3. Martínez-García E, Aparicio T, Goñi-Moreno A, Fraile S, de Lorenzo V. 2015. SEVA 2.0: an update of the Standard European Vector Architecture for de-/re-construction of bacterial functionalities. Nucleic Acids Res 43:D1183–D1189.
4. Weber E, Engler C, Gruetzner R, Werner S, Marillonnet S. 2011. A Modular Cloning System for Standardized Assembly of Multigene Constructs. PLoS One 6:e16765.
5. Blázquez B, Torres-Bacete J, San Leon D, Kniewel R, et al. (2022) Golden Standard: A complete standard, portable, and interoperative MoClo tool for model and non-model bacterial hosts https://doi.org/10.1101/2022.09.20.508659.
